# Socio-economic status and ethnicity are independently associated with dietary patterns: the HELIUS-Dietary Patterns study

**DOI:** 10.3402/fnr.v59.26317

**Published:** 2015-06-02

**Authors:** Louise H. Dekker, Mary Nicolaou, Rob M. van Dam, Jeanne H. M. de Vries, Evelien J. de Boer, Henny A. M. Brants, Marja H. Beukers, Marieke B. Snijder, Karien Stronks

**Affiliations:** 1 Department of Public Health, Academic Medical Centre, University of Amsterdam, Amsterdam, The Netherlands; 2 Saw Swee Hock School of Public Health and Department of Medicine, Yong Loo Lin School of Medicine, National University of Singapore and National University Health System, Singapore; 3 Division of Human Nutrition, Wageningen University, Wageningen, The Netherlands; 4 Centre for Nutrition, Prevention and Health Services, National Institute for Public Health and the Environment (RIVM), Bilthoven, The Netherlands

**Keywords:** dietary patterns, non-Western ethnic minority groups, education, occupation, socio-economic status, HELIUS study

## Abstract

**Background:**

Differences in dietary patterns between ethnic groups have often been observed. These differences may partially be a reflection of differences in socio-economic status (SES) or may be the result of differences in the direction and strength of the association between SES and diet.

**Objective:**

We aimed to examine ethnic differences in dietary patterns and the role of socio-economic indicators on dietary patterns within a multi-ethnic population.

**Design:**

Cross-sectional multi-ethnic population-based study.

**Setting:**

Amsterdam, the Netherlands.

**Subjects:**

Principal component analysis was used to identify dietary patterns among Dutch (*n*=1,254), South Asian Surinamese (*n*=425), and African Surinamese (*n*=784) participants. Levels of education and occupation were used to indicate SES. Linear regression analysis was used to examine the association between ethnicity and dietary pattern scores first and then between socio-economic indicators and dietary patterns within and between ethnic groups.

**Results:**

‘Noodle/rice dishes and white meat’, ‘red meat, snacks, and sweets’ and ‘vegetables, fruit and nuts’ patterns were identified. Compared to the Dutch origin participants, Surinamese more closely adhered to the ‘noodle/rice dishes and white meat’ pattern which was characterized by foods consumed in a ‘traditional Surinamese diet’. Closer adherence to the other two patterns was observed among Dutch compared to Surinamese origin participants. Ethnic differences in dietary patterns persisted within strata of education and occupation. Surinamese showed greater adherence to a ‘traditional’ pattern independent of SES. Among Dutch participants, a clear socio-economic gradient in all dietary patterns was observed. Such a gradient was only present among Surinamese dietary oatterns to the ‘vegetables, fruit and nuts’ pattern.

**Conclusions:**

We found a selective change in the adherence to dietary patterns among Surinamese origin participants, presumably a move towards more vegetables and fruits with higher SES but continued fidelity to the traditional diet.

In European countries, the immigrant population is growing enormously. Differences in diet between ethnic minority groups and host populations have been observed (1–4), in which the preservation of traditional dietary habits is seen (5–7), but also adoption of the Western diet, high in meat and fat intake, is shown (5–8). The adoption of the Western diet has been suggested to increase the risk of adverse health effects such as obesity and type 2 diabetes (9), also among ethnic minority groups (10). To fully understand the differences in diet, studying the overall dietary pattern rather than single nutrients or foods has been suggested (11). There is a need to describe these patterns of food consumption and their determinants in order to better understand the role of diet in the observed ethnic inequalities in health. This understanding is key in formulating strategies to stimulate healthy diet as dietary change may be more readily achieved when recommended foods are compatible with existing patterns of food consumption (12).

Differences in dietary patterns between ethnic groups may partially be a reflection of differences in socio-economic status (SES). Among non-migrant populations, there is strong evidence that dietary patterns are more favourable in higher SES individuals (13–17). Ethnic minorities are often overrepresented in low SES groups, implying that differences in their dietary patterns are a reflection of differences in their socio-economic profile.

To date, there is little evidence as to whether the well-known SES gradient in diet also applies to ethnic minority groups. The direction and strength of the association between SES and diet might differ across ethnic groups (18) because of the complex interaction between diet and ethnicity; diet is governed by deeply rooted cultural norms and values, and has particular significance for ethnic and cultural identity. Some aspects of the diet may be relinquished or adopted more readily than others (19), which may lead to a different association with SES as compared to the host population. It is, therefore, important to assess the role of SES in dietary patterns before potential differences are wrongly assigned to ethnic origin.

Therefore, this paper aims to describe the dietary patterns of one of the largest ethnic minority groups in the Netherlands (South Asian and African origin Surinamese) in comparison with the majority, Dutch origin, population and to investigate to what extent SES characteristics of these populations contribute to differences in dietary patterns both *between* and *within* ethnic groups.

## Methods

### Study design and subjects

Participants were recruited within the HEalthy LIfe in an Urban Setting (HELIUS) study, and detailed information can be found elsewhere (20, 21). In brief, the HELIUS study was designed as a prospective cohort study and is being carried out in Amsterdam, the Netherlands. The primary aim of HELIUS is to unravel the causes of the unequal burden of disease across ethnic groups and includes people of Dutch, African Surinamese, South Asian Surinamese, Turkish, Moroccan, and Ghanaian origin. Together, these groups are the largest ethnic groups in Amsterdam. Adults aged 18–70 years are randomly sampled, stratified by ethnic origin, through the municipal registry of Amsterdam.

Ethnic origin was defined on the basis of country of birth: persons were defined as of non-Dutch origin if she/he fulfilled one of two criteria: born outside the Netherlands and has at least one parent also born outside the Netherlands (first generation); or born in the Netherlands but both parents were born outside the Netherlands (second generation) (22). In this paper, we focus on South Asian Surinamese, African Surinamese, and Dutch origin participants.

For this study, we included a sub-sample of the HELIUS population; participants that consented to being approached for additional studies were eligible to participate in this sub-study (the HELIUS-Dietary Patterns study). Baseline HELIUS data collected until June 2013 were used.

Data on population characteristics and health status were collected through a questionnaire/interview and a physical examination. Biological samples were obtained during the physical examination. The HELIUS study was ethically approved by the AMC Medical Ethics Committee.

### Food frequency questionnaire

Dietary intake was collected using ethnic-specific semi-quantitative food frequency questionnaires (FFQs) with a reference period of 4 weeks. An existing validated Dutch FFQ (23) formed the basis for a new comparable FFQ, specifically assessing dietary intake in the Surinamese population. Detailed information about the FFQ development can be found elsewhere (24). In brief, data from single 24-hour recalls, collected within a Surinamese population, formed the basis for the adaptation of this FFQ, using a standardized approach (25). Food items were selected according to their percentage contribution to, and variance in nutrient intake. A nutrient database was constructed consisting of data of the Dutch Food Composition table 2011 (26). Data on ethnic-specific foods was based on new chemical analyses, and available international data. The FFQ included ~220 food items covering more than 90% of the intake of the main nutrients of interest.

### Assessment of dietary patterns

Dietary patterns were derived on the basis of principal components analysis (PCA), which assesses the correlations between food groups to identify the underlying patterns in the data. This approach allows assessment of the whole diet, accounting for the fact that foods/nutrients are consumed in combination and are therefore highly correlated.

Intakes of food groups were obtained by collapsing food items assessed in the FFQ on the basis of similarity in nutrient profile, culinary use, or ethnic origin (Supplementary Table 1). Some ethnic-specific foods were not combined within a broader food group category in order to prevent loss of possibly relevant details, e.g. roti (Indian flat bread) only assessed in the Surinamese FFQ was not combined with the low fibre bread food group because of a distinctive culinary use. This resulted in a total of 49 food groups: 35 food groups consisted of the same foods in all ethnic groups; 11 food groups were variable in content because of the inclusion of ethnic-specific items, e.g. Asian sweets in the cakes and cookies group; 1 food group was applicable to the Dutch FFQ (pancakes), and 2 food groups were applicable to the Surinamese FFQ only (roti and pom).

To describe differences in dietary patterns between ethnic groups, we performed PCA for the whole sample (i.e. ethnic Dutch, South Asian Surinamese, and African Surinamese combined). The dietary patterns were derived on the basis of the unadjusted consumption (g/day) of each food group. The number of components retained was based on the following criteria: components with an eigenvalue >1, Scree plot test, and the interpretability of the components. Food items were considered to load on a component if they had an absolute factor loading ≥0.3. A larger factor loading indicates a higher correlation of the food group to the respective component. We report the percentage of variance of the food group intake explained by each pattern. This aspect, however, did not play a role in the selection of components as it depends highly on the number of variables included in the analysis. The Scree plot test clearly identified three major components (hereafter called ‘dietary patterns’). For ease of describing each of these dietary patterns, we labelled them on the basis of food groups that loaded highest.

Each participant received a factor score for each dietary pattern that emerged, calculated by summing the standardized intake of foods, weighted by the factor loadings of the foods groups for each dietary pattern. These scores rank individuals according to the degree to which they adhered to each of the derived dietary patterns. We calculated partial correlation coefficients, adjusted for energy intake, between nutrient intake and dietary pattern scores in order to get insight into the macronutrient composition of each of the dietary patterns.

To understand the consequences of performing pooled analysis, we repeated our analysis with the study population stratified on the basis of sex and ethnicity. Furthermore, we examined the influence of the unequal distribution of ethnicities on the derived dietary patterns by running the analysis with a random sample of *n*=200 per ethnic group.

### Assessment of SES

Educational and occupational levels were used as proxies for SES. Educational level was indicated by the highest education attained. Participants were categorized into: ‘never been to school, elementary schooling, lower vocational schooling, or lower secondary schooling’, ‘intermediate vocational schooling or intermediate/higher secondary schooling (general)’, and ‘higher vocational schooling or university’. These levels are subsequently referred to in the models as low, medium, or high. Current occupational status was based on three different levels. The ‘lowest’ class represented occupations characterized by ‘manual labour’ (skilled and unskilled manual), followed by the ‘middle class’ characterized by ‘lower grade professionals and routine non-manual labour’, and the ‘highest’ occupational level characterized by ‘higher grade professionals’.

### Other variables

Smoking was assessed as current smoker, former smoker, or never smoked. Alcohol intake was assessed as currently using alcohol or not. Marital status consisted of two categories, either married/living with a partner or not (never married, divorced or separated, or widow/widower). Participants were classified as having diabetes on the basis of fasting glucose ≥7 mmol/l or using glucose lowering medication. Hypertension was based WHO-criteria (SBP ≥140 mmHg and DBP ≥90 or use of blood pressure lowering medication). Hypercholesterolemia was defined as total serum cholesterol ≥6.2 mmol/l (240 mg/dl) or using lipid lower medication. The presence of disease ‘morbidity’ variable included participants that were coded as having at least one of either diabetes, hypertension, or hypercholesterolemia.

### Statistical analysis

Baseline characteristics were expressed as percentages (%), or means with standard deviations (SD). Linear regression analysis was used to examine the associations between ethnicity and dietary pattern scores, where 1 unit change of each score corresponded to 1 SD of the study population. Distribution of continuous variables was examined for normality and log-transformed when necessary before entering them into the regression models. Due to a significant interaction between ethnicity and sex in the association with dietary patterns, we conducted analysis separately for men and women. In addition to the age-adjusted model (Model 1), we adjusted for marital status, morbidity, smoking status, physical activity, and body mass index (BMI) (Model 2). To understand the role of SES within the different ethnic groups (i.e. the SES gradient in dietary patterns), we studied the association between socio-economic indicators and dietary pattern scores within ethnic groups. Additionally, we tested for interaction by SES in the association between ethnicity and dietary pattern scores. All analyses were performed with SPSS version 20 (IL, USA).

## Results

### Baseline characteristics

Table 1 presents selected characteristics of the study population by ethnicity. The average age of the participants was 49 years, and there were considerably more women than men in all ethnic groups; the percentage of current smokers was similar across ethnic groups (~23%). Compared to the other groups, African Surinamese were more often unmarried, divorced, or widowed (67.2%). Dutch more often reported using alcohol (92.1%), had the lowest BMI (mean: 24.8 kg/m^2^), and had the highest proportion of highly educated participants and participants working at the highest occupational level (60.1 and 57.8%, respectively). More Surinamese participants had diabetes, hypertension or elevated cholesterol, with diabetes being most prevalent among South Asian Surinamese (19.1%) and hypertension most prevalent among African Surinamese (56.4%) (data not shown).

**Table 1:** Population characteristics of the HELIUS-Dietary Patterns study

		Dutch	South Asian Surinamese	African Surinamese
		(*n*=1,254)	(*n*=425)	(*n*=784)
Age (mean, SD)		48.3 (13)	47.9 (12)	49.7 (11)
Sex, %	Men	45.6	43.3	33.4
Smoking, %	Yes	23.1	23.5	23.5
	Never smoked	37.7	62.1	54.1
	Past smoker	39.0	14.3	22.1
Alcohol intake, %	Yes	92.1	53.3	65.4
Educational level, %	Low	17.4	49.5	36.3
	Middle	22.5	26.2	35.3
	High	60.1	24.1	28.4
Occupational status^a^, %	Low	15.6	49.1	35.8
	Middle	26.6	28.7	38.7
	High	57.8	22.0	25.5
Marital status, %	Married/registered partnership or cohabiting (living together)	60.0	53.2	32.8
	Unmarried (never married), divorced or separated, or widow/widower	40.0	46.8	67.2
Generation status, %	First	–	84.4	89.0
	Second	–	15.6	11.0
Years in the Netherlands (first generation), %		–	34.0	32.1
Morbidity, yes %		39.8	56.1	60.3
Body mass index (BMI) in kg/m^2^, mean (SD)		24.8 (4.0)	26.3 (4.7)	28.1 (5.6)

a There were 177 missing cases with respect to occupational status, percentage of missing cases did not differ significantly between the ethnic groups.

### Dietary patterns

Within each ethnic group, we extracted comparable dietary patterns (in all three groups, we derived a clear meat, a snack, and a vegetable pattern), suggesting that the data could be pooled in order to describe differences in dietary patterns between the ethnic groups. For this pooled analysis, the factor loadings ≥0.30 of food groups for the three identified dietary patterns are shown in Table 2. Positive factor loadings indicate that the subsequent food group is highly correlated with the respective dietary pattern. The ‘noodle/rice dishes and white meat’ pattern was characterized by high intakes of rice and noodles dishes, chicken, organ meat, fish, savoury bread filling, savoury sauces, sugar sweetened beverages, low fibre bread and bread products, and Surinamese dishes like pom (Surinamese festive dish) and roti. Most of these foods are typically consumed in a traditional Surinamese diet. The second pattern, labelled as the ‘red meat, snacks, and sweets’ pattern, was characterized by higher intakes of red meat and processed meat, pasta, snacks, sugar and sweets, French fries, beer, cheese, fat and oil (not olive oil) and full fat margarine, savoury sauces, cakes and cookies, potatoes and other root vegetables, pancakes, and high fibre bread and bread products. Food groups in the third pattern, the so-called ‘vegetables, fruit and nuts pattern’, had high factor loadings on meat substitutes and other soy products, nuts and seeds, tomato and tomato products, brassica vegetables, other vegetables, legumes, olive oil, fruit and low fat fish. Each pattern explained approximately 6% of the total variation in food group intake data. There was no confounding by seasonal differences in the time point of dietary assessment (data not shown) (*P*=0.45).

**Table 2:** Factor loadings of dietary patterns among Dutch, South Asian Surinamese, and African Surinamese origin participants of the HELIUS-Dietary Patterns study

	Factor loadings		
	
Food groups	Noodle/rice dishes and white meat pattern	Red meat, snacks, and sweets pattern	Vegetables, fruit, and nuts pattern
Rice and noodle dishes	**0.64**	−0.06	−0.23
Chicken	**0.63**	0.04	−0.13
Low fat fish	**0.52**	−0.13	**0.31**
Indian flat bread (roti)	**0.47**	−0.13	−0.02
Savoury bread fillings	**0.44**	0.11	0.09
High fat fish	**0.42**	−0.11	0.26
Sugar sweetened beverages	**0.42**	0.24	−0.26
Pom	**0.42**	−0.08	0.11
Savoury sauces	**0.41**	**0.38**	0.10
Low fibre bread and bread products	**0.38**	0.22	−0.26
Coffee	−**0.36**	0.14	0.28
Wine, sherry, port, vermouth	−**0.35**	0.20	0.16
Organ meat	**0.33**	0.11	0.08
Red meat	0.13	**0.57**	0.04
Snacks (fried snacks, potato chips, salty snacks)	0.11	**0.56**	−0.06
Processed meat	−0.04	**0.52**	−0.01
Sugar and sweets	0.16	**0.47**	−0.03
Pasta	−0.10	**0.46**	0.13
Cakes and cookies	0.06	**0.44**	0.06
French fries and other fried potato dishes	0.28	**0.42**	−0.15
Potatoes and other root vegetables	0.05	**0.41**	0.13
Cheese	−0.09	**0.40**	0.11
Pancakes	−0.22	**0.40**	0.06
Fat, oil (not olive oil), and full fat margarines	0.17	**0.35**	0.03
Beer	−0.09	**0.30**	−0.02
High fibre bread and bread products	−0.09	**0.30**	0.20
‘Other’ vegetables	−0.04	−0.07	**0.78**
Tomato and tomato products	−0.10	0.04	**0.61**
Brassica vegetables	−0.16	0.04	**0.58**
Olive oil	−0.11	0.13	**0.49**
Fruit	0.20	−0.20	**0.45**
Nuts and seeds	0.07	0.14	**0.41**
Meat substitutes and other soy products	−0.15	−0.03	**0.33**
Legumes	0.28	0.06	**0.32**
Explained variance (%)	6.8	6.8	6.3

Food groups with factor loadings ≥0.30 for one of the dietary patterns.

Higher scores on the noodle/rice dishes and white meat pattern and on the red meat, snacks, and sweets pattern were significantly associated with higher intakes of total energy in all three ethnic groups (Table 3). The red meat, snacks, and sweets pattern was negatively associated with intakes of non-haem iron, vitamin C and, particularly, among Dutch origin participants, there was a strong negative correlation with dietary fibre (−0.46). Higher scores on the vegetables, fruit, and nuts pattern were associated with significantly lower intakes of carbohydrates, particularly among the Surinamese groups. Strong positive associations were observed between the vegetables, fruit and nuts pattern scores, and dietary fibre, iron, β–carotene, and vitamin C intakes.

**Table 3:** Partial Pearson correlation coefficients (r) between each of three food patterns derived in the HELIUS-Dietary Patterns study and daily energy and nutrient intakes

	Noodle/rice dishes and white meat pattern				Red meat, snacks, and sweets pattern				Vegetables, fruit and nuts pattern			
	
	Total group	Ethnic Dutch	South Asian Surinamese	African Surinamese	Total group	Ethnic Dutch	South Asian Surinamese	African Surinamese	Total group	Ethnic Dutch	South Asian Surinamese	African Surinamese
Energy (kcal)	**0.42**	**0.37**	**0.76**	**0.71**	**0.71**	**0.83**	**0.75**	**0.71**	**0.25**	0.18	**0.30**	**0.24**
Carbohydrates	**0.24**	0.13	−0.08#	−0.06#	−**0.22**	−0.09	0.02	−0.03	−**0.34**	−0.10	−**0.42**	−**0.47**
Fibre	−**0.20**	−0.13	−0.04#	−0.15	−0.19	−**0.46**	−**0.30**	−**0.29**	**0.61**	**0.63**	**0.57**	**0.58**
Protein	**0.22**	0.19	**0.24**	**0.26**	−0.14	−0.01#	−**0.24**	−0.15	**0.27**	0.15	**0.39**	**0.38**
Total fatty acids	−0.19	0.00#	0.00#	−0.03#	**0.24**	0.15	0.09#	0.12	**0.21**	−0.03#	**0.26**	**0.34**
SFA	−**0.38**	−0.16	−0.13	−0.11	**0.43**	**0.26**	**0.26**	0.30	−0.03#	−**0.24**	−0.07#	0.00
MUFA	−0.12	0.13	0.05#	−0.03#	0.20	0.17	0.03#	0.07	**0.24**	−0.05#	**0.34**	**0.42**
PUFA	**0.20**	0.08	0.09	0.07	−0.19	−0.11	−0.10	−0.10	**0.27**	**0.29**	**0.31**	**0.39**
Alcohol	−**0.41**	−**0.24**	−0.16	−0.04	**0.26**	−0.02#	0.06#	0.06#	0.15	0.00#	0.08#	0.07#
Calcium	−**0.25**	−0.16	−0.08#	−0.06#	0.02#	−**0.21**	−**0.24**	−0.07	**0.32**	0.17	**0.41**	**0.35**
Total iron	**0.22**	−0.09	0.16	0.06#	0.04	−**0.28**	−0.11	−0.11	**0.62**	**0.60**	**0.61**	**0.59**
Iron non–haem	−**0.25**	−0.19	0.09	0.01#	−0.04#	−**0.40**	−0.15	−0.17	**0.65**	**0.66**	**0.64**	**0.61**
Iron haem	0.10	**0.26**	**0.23**	**0.26**	**0.23**	**0.39**	0.07#	0.15	−0.11	−**0.25**	−0.02#	−0.05#
β-carotene	−0.04	−0.03#	0.08#	−0.03	−**0.20**	−**0.34**	−**0.26**	−**0.22**	**0.65**	**0.71**	**0.65**	**0.60**
Vitamin C	0.07	0.16	0.13	0.10	−**0.21**	−**0.33**	−**0.37**	−**0.23**	**0.46**	**0.52**	**0.48**	**0.39**
Vitamin D	**0.24**	**0.27**	0.14	**0.22**	−0.12	0.11	−0.09#	−0.16	0.08	−0.02#	0.11	**0.24**

Except for total energy intake, nutrients are adjusted for energy intake; all nutrients are log-transformed to improve normality. All correlations are statistically significant, except for those correlations marked with #; values in bold indicate partial Pearson correlations ≥0.20; SFA, saturated fatty acids; MUFA, mono unsaturated fatty acids; PUFA, poly unsaturated fatty acids.

### Ethnic differences in dietary pattern scores

Table 4 shows ethnic differences in dietary pattern scores for each of the dietary patterns. We extracted similar patterns for men and women; however, we found significant interaction by sex with regard to ethnic differences in pattern scores (*p*≤0.001). Therefore, the results are displayed separately for men and women. Compared to Dutch men, in the fully adjusted model, Surinamese men had significantly higher scores on the noodle/rice dishes and white meat (β (95% confidence interval, CI): 1.33 (1.19; 1.48) and 1.35 (1.22; 1.47) for the South Asian and African Surinamese men, respectively), and significant lower scores for the red meat, snacks, and sweets pattern and the vegetables, fruit, and nuts dietary patterns, adjusted for potential confounders. These differences in pattern scores were greatest among men, especially with respect to the adherence to the noodle/rice dishes and white meat dietary pattern. Compared to the first model, adding marital status, morbidity, smoking status, and physical activity did not significantly change our results, this, in contrast to BMI. Therefore, in the interest of statistical power, we decided to adjust all following analysis for age and BMI only.

**Table 4:** Ethnic differences in dietary pattern scores derived in the HELIUS-Dietary Patterns study

Dietary pattern	Noodle/rice dishes and white meat pattern	Red meat, snacks, and sweets pattern	Vegetables, fruit and nuts pattern	Noodle/rice dishes and white meat pattern	Red meat, snacks, and sweets pattern	Vegetables, fruit and nuts pattern
	Women			Men		
Model 1	β (95% CI)					
Dutch	Ref.	Ref.	Ref.	Ref.	Ref.	Ref.
South Asian Surinamese	1.17 (1.06; 1.27)*	−0.87 (−0.98; −0.76)*	−0.37 (−0.52; −0.23)*	1.35 (1.21; 1.48)*	−1.20 (−1.35; −1.05)*	−0.58 (−0.74; −0.42)*
African Surinamese	1.13 (1.05; 1.21)*	−0.65 (−0.74; −0.57)*	−0.38 (−0.49; −0.27)*	1.39 (1.27; 1.51)*	−0.93 (−1.06; −0.79)*	−0.54 (−0.69; −0.40)*
Model 2						
Dutch	Ref.	Ref.	Ref.	Ref.	Ref.	Ref.
South Asian Surinamese	1.10 (0.99; 1.21)**	−0.89 (−1.00; −0.78)*	−0.27 (−0.41; −0.12)*	1.33 (1.19; 1.48)*	−1.22 (−1.38; −1.07)*	−0.49 (−0.66; −0.33)*
African Surinamese	1.02 (0.92; 1.11)*	−0.65 (−0.74; −0.55)*	−0.28 (−0.41; −0.16)*	1.35 (1.22; 1.47)*	−0.99 (−1.13; −0.85)*	−0.48 (−0.62; −0.32)*

* *p*≤0.001. Model 1: adjust for age; Model 2: model 1+marital status, morbidity, smoking status, physical activity and BMI. Regression coefficients reflect the difference between the dietary pattern scores within each ethnic group compared to the reference groups (ethnic Dutch).

### Ethnic differences in dietary pattern scores according to level of SES

When adding an interaction term to the model, we observed significant interaction between ethnicity and SES. Therefore, the results are presented stratified by SES. Across socio-economic groups, ethnic differences in dietary pattern scores were robust. Within most education or occupation levels, we observed significant ethnic differences in dietary pattern scores (Table 5). Among women, both Surinamese groups adhered less to the vegetables, fruit, and nuts pattern compared to the Dutch, although the differences were only statistically significant in the highly educated Surinamese (β (95% CI): −0.38 (−0.67; −0.09) and −0.31 (−0.50; −0.12) for the South Asian and African Surinamese, respectively). This was also observed within the levels of occupational status, although differences in adherence to the vegetables, fruit, and nuts pattern were not statistically significant between the Dutch and the South Asian Surinamese at all occupation levels. Among men, ethnic differences in dietary patterns were consistent within educational and occupational level strata.

**Table 5:** The association between ethnicity and dietary pattern scores by socio-economic status in the HELIUS-Dietary Patterns study

		Noodle/rice dishes and white meat pattern	Red meat, snacks, and sweets pattern	Vegetables, fruit and nuts pattern		Noodle/rice dishes and white meat pattern	Red meat, snacks, and sweets pattern	Vegetables, fruit and nuts pattern
	Women				Men			
Education^a^	*n*	β (95% CI)			*n*	β (95% CI)		
Low								
Dutch	121	Ref.	Ref.	Ref.	97	Ref.	Ref.	Ref.
South Asian Surinamese	119	0.86 (0.67; 1.06)*	−1.18 (−1.36; −1.01)*	−0.02 (−0.25; 0.21)	91	1.11 (0.82; 1.40)*	−1.58 (−1.84; −1.33)*	−0.33 (−0.60; −0.06)*
African Surinamese	165	0.87 (0.68; 1.06)*	−0.94 (−1.10; −0.78)*	−0.17 (−0.38; 0.04)	118	1.17 (0.90; 1.45)*	−1.27 (−1.51; −1.02)*	−0.23 (−0.48; 0.01)
Middle								
Dutch	149	Ref.	Ref.	Ref.	132	Ref.	Ref.	Ref.
South Asian Surinamese	69	1.26 (1.03; 1.49)*	−0.82 (−1.04; −0.59)*	−0.07 (−0.33; 0.18)	42	1.28 (0.95; 1.61)*	−1.41 (−1.72; −1.09)*	−0.40 (−0.76; −0.04)*
African Surinamese	189	0.95 (0.77; 1.14)*	−0.73 (−0.90; −0.55)*	−0.18 (−0.38; 0.01)	86	1.34 (1.08; 1.60)*	−1.18 (−1.42; −0.94)*	−0.43 (−0.71; −0.16)*
High								
Dutch	410	Ref.	Ref.	Ref.	342	Ref.	Ref.	Ref.
South Asian Surinamese	51	1.12 (0.95; 1.29)*	−0.79 (−1.01; −0.57)*	−0.38 (−0.67; −0.09)*	51	1.34 (1.16; 1.53)*	−1.09 (−1.35; −0.83)*	−0.53 (−0.82; −0.25)*
African Surinamese	167	1.12 (1.00; 1.23)*	−0.57 (−0.71; −0.42)*	−0.31 (−0.50; −0.12)*	167	1.26 (1.08; 1.44)*	−0.88 (−1.13; −0.62)*	−0.52 (−0.81; −0.25)*
Occupation^a^								
Low								
Dutch	105	Ref.	Ref.	Ref.	80	Ref.	Ref.	Ref.
South Asian Surinamese	99	1.02 (0.81; 1.24)*	−1.19 (−1.38; −1.00)*	−0.15 (−0.39; 0.10)	89	1.12 (0.78; 1.48)*	−1.61 (−1.89; −1.33)*	−0.41 (−0.72; −0.11)*
African Surinamese	136	0.90 (0.69; 1.12)*	−0.93 (−1.09; −0.75)*	−0.42 (−0.65; −0.19)*	121	1.40 (1.07; 1.73)*	−1.21 (−1.47; −0.95)*	−0.29 (−0.58; −0.01)*
Middle								
Dutch	175	Ref.	Ref.	Ref.	140	Ref.	Ref.	Ref.
South Asian Surinamese	71	1.01 (0.80; 1.21)*	−0.81 (−1.02; −0.60)*	−0.23 (−0.49; 0.02)	39	1.42 (1.18; 1.66)*	−1.35 (−1.65; −1.06)*	−0.41 (−0.71; −0.11)*
African Surinamese	211	0.99 (0.84; 1.15)*	−0.69 (−0.84; −0.54)*	−0.21 (−0.39; −0.02)*	67	1.06 (0.86; 1.26)*	−1.34 (−1.62; −1.15)*	−0.31 (−0.55; −0.06)*
High								
Dutch	364	Ref.	Ref.	Ref.	322	Ref.	Ref.	Ref.
South Asian Surinamese	46	1.23 (1.05; 1.40)*	−0.79 (−1.02; −0.55)*	−0.31 (−0.61; 0.02)	38	1.35 (1.46; 1.55)*	−1.10 (−1.38; −0.82)*	−0.33 (−0.66; 0.01)
African Surinamese	132	1.12 (0.01; 1.24)*	−0.58 (−0.74; −0.43)*	−0.22 (−0.42; −0.02)*	51	1.25 (1.07; 1.43)*	−0.79 (−1.05; −0.54)*	−0.57 (−0.87; −0.27)*

Sex-specific linear regression models adjusted for age and BMI stratified by education or occupation. Regression coefficients reflect the difference between the dietary pattern scores within each ethnic group compared to the reference groups (Dutch origin).

* *p*≤0.001.

a There are 10 missing cases on educational level. There are 177 missing cases with respect to occupational status. These missing cases are not significantly different between the ethnic groups.

### The socio-economic gradient of dietary pattern scores within each ethnic group

We observed a clear SES gradient in pattern scores within the Dutch origin population (Table 6). As expected, higher educational and occupational levels were associated with lower scores for the noodle/rice dishes and white meat pattern and the red meat, snacks, and sweets pattern, and higher scores for the vegetables, fruit, and nuts pattern. Within both Surinamese groups, a socio-economic gradient was most consistently seen with respect to the vegetables, fruit, and nuts pattern, although non-significant among African Surinamese men. The higher the occupational level of African Surinamese men, the lesser they adhered to the noodle/rice dishes and the white meat pattern; this pattern was not observed in South Asian Surinamese men and Surinamese women.

**Table 6:** The association between socio-economic status and dietary patterns within sex-specific ethnic groups in the HELIUS-Dietary Patterns study

		Noodle/rice dishes and white meat pattern	Red meat, snacks, and sweets pattern	Vegetables, fruit and nuts pattern		Noodle/rice dishes and white meat pattern	Red meat, snacks, and sweets pattern	Vegetables, fruit and nuts pattern
	Women				Men			
Education^a^	n	B (95% CI)			n	B (95% CI)		
Dutch								
Low	121	Ref.	Ref.	Ref.	97	Ref.	Ref.	Ref.
Middle	149	−0.09 (−0.19; 0.01)	−0.02 (−0.20; 0.16)	0.17 (−0.04; 0.38)	132	−0.19 (−0.26; 0.01)	−0.10 (−0.34; 0.15)	0.19 (−0.05; 0.43)
High	410	−0.27** (−0.36;−0.18)	−0.18** (−0.34; −0.02)	0.60** (0.41; 0.79)	342	−0.22** (−0.34; −0.10)	−0.43** (−0.65; −0.22)	0.51** (0.29; 0.73)
South Asian Surinamese								
Low	119	Ref.	Ref.	Ref.	91	Ref.	Ref.	Ref.
Middle	69	0.34* (0.06; 0.62)	0.18 (−0.03; 0.41)	0.20 (−0.08; 0.49)	42	−0.11 (−0.53; 0.32)	−0.13 (−0.45; 0.49)	0.16 (−0.22; 0.54)
High	51	−0.05 (−0.36; 0.26)	−0.05 (−0.30; 0.17)	0.36* (0.04; 0.68)	51	−0.13 (−0.52; 0.25)	−0.12 (−0.41; 0.18)	0.36* (0.02; 0.71)
African Surinamese								
Low	165	Ref.	Ref.	Ref.	118	Ref.	Ref.	Ref.
Middle	189	0.02 (−0.26; 0.01)	0.08 (−0.07; 0.25)	0.25* (0.04; 0.47)	86	−0.11 (−0.43; 0.21)	−0.22 (−0.45; 0.02)	0.03 (−0.27; 0.34)
High	167	−0.06 (−0.25; 0.12)	0.08 (−0.07; 0.24)	0.51** (0.29; 0.73)	54	−0.26 (−0.64; 0.11)	−0.18 (−0.45; 0.09)	0.24 (−0.11; 0.59)
Occupation^a^								
Dutch								
Low	105	Ref.	Ref.	Ref.	80	Ref.	Ref.	Ref.
Middle	175	−0.08 (−0.18; 0.02)	−0.10 (−0.27; 0.07)	0.14 (−0.07; 0.35)	140	−0.12 (−0.26; 0.01)	−0.10 (−0.35; 0.14)	−0.13 (−0.11; 0.38)
High	364	−0.22** (−0.31; −0.13)	−0.24** (−0.41; −0.09)	0.44** (0.25; 0.63)	322	−0.26** (−0.38; −0.14)	−0.56** (−0.78; −0.35)	−0.47** (0.26; 0.70)
South Asian Surinamese								
Low	99	Ref.	Ref.	Ref.	89	Ref.	Ref.	Ref.
Middle	71	−0.06 (−0.35; 0.21)	0.24* (0.02; 0.46)	0.06 (−0.23; 0.35)	39	0.12 (−0.29; 0.53)	−0.09 (−0.20; 0.39)	0.17 (−0.19; 0.54)
High	46	−0.05 (−0.38; 0.25)	0.02 (−0.23; 0.28)	0.33 (−0.01; 0.66)	38	−0.08 (−0.51; 0.33)	−0.17 (−0.47; 0.13)	0.60* (0.22; 0.98)
African Surinamese								
Low	136	Ref.	Ref.	Ref.	121	Ref.	Ref.	Ref.
Middle	211	0.07 (−0.11; 0.26)	−0.13 (−0.03; 0.29)	0.35** (0.13; 0.57)	67	−0.48** (−0.82; −0.14)	−0.31* (−0.55; −0.05)	0.13 (−0.19; 0.45)
High	132	−0.04 (−0.25; 0.17)	0.08 (−0.09; 0.26)	0.64** (0.41; 0.89)	51	−0.42* (−0.80; −0.05)	−0.17 (−0.44; 0.10)	0.23 (−0.12; 0.58)

Sex-specific linear regression models adjusted for age and BMI stratified by education or occupation.

* *p*≤0.001.

** *p*≤0.001.

a There are 10 missing cases on educational level. There are 177 missing cases with respect to occupational status. The percentage of missing cases are not significantly different between the ethnic groups.

## Discussion

Three major dietary patterns were identified, labelled as a ‘noodle/rice dishes and white meat’; ‘red meat, snacks, and sweets’; and ‘vegetables, fruit and nuts’ pattern. Surinamese origin participants clearly showed greater adherence to the noodle/rice dishes and white meat, while Dutch origin participants scored significantly higher on the other two dietary patterns. Overall, these ethnic differences in pattern scores were robust within different SES groups, with the greatest ethnic differences in dietary pattern scores among men. A SES gradient was observed in Dutch origin participants, with higher adherence to the vegetable, fruit, and nut pattern and lower adherence to the red meat, snacks, and sweets pattern in participants of higher SES. Among Surinamese, this gradient was only observed with regard to adherence to the vegetables, fruit, and nuts pattern and differed between SES indicators and between men and women.

Few studies have investigated the dietary patterns of different ethnic groups living in one setting. These studies found that some patterns are shared by different ethnic groups (often a ‘Western’ pattern (high in snacks and meat) and a ‘healthy’ pattern (high in vegetables, fruits, and fish), whereas other patterns are ethnic specific (1, 27–29). In our sample, we found similar patterns, including an ‘ethnic-specific’ pattern (the noodle/rice dishes and white meat pattern), that more closely resembled a ‘traditional’ Surinamese pattern. This study adds additional insight by considering the association between dietary patterns with SES. We found that adherence to the ethnic-specific pattern was quite robust across different occupational and educational groups.

Both the noodle/rice dishes and white meat pattern and the red meat, snacks, and sweets pattern were characterized by high intakes of presumably ‘less healthy’ food groups (i.e. red meat, snacks and French fries, sugar sweetened beverages, and savoury sauces). This was further underscored by the highly positive correlations between these patterns and total energy intake, saturated fatty acids, and the highly negative correlations with fibre, β–carotene, and vitamin C. One would expect to see a decrease in adherence to these patterns with increasing SES in all ethnic groups. However, this gradient was only observed in the Dutch origin group. The high adherence to the noodle/rice dishes and white meat pattern (a more traditional Surinamese pattern), regardless of SES in combination with higher adherence to the vegetables, fruit, and nuts pattern to their diet with higher SES indicates a selective adoption of diet. Interesting in this context, Sharma and Cruickshank (30) reported that African Caribbean adults in Britain, despite their low incomes, spent more on traditional foods like yams than on potatoes, thereby maintaining cultural food preferences. This suggests that ethnic minority/migrant origin groups assign a certain importance to foods closely associated with their traditional dietary patterns.

Among ethnic minority groups, the expected change in dietary behaviour with increasing SES might be complicated by the value that is given to traditional foods, as described by the model of Kocturk-Runefors (31). Foods that are strongly associated with cultural identity, values, and norms (i.e. staple foods as rice and bread) may be the last to change. Whereas accessory foods (i.e. vegetables, meat, and chicken) or ‘extras’ (i.e. fruits, sweets, and nuts) are less valued and change in their use seems more often related to availability or the economic situation. In this study, foods characterizing the vegetable, fruits, and nuts pattern are presumably less associated with cultural identity; thus, it is easier to adopt these foods than those foods that characterize the noodle/rice dishes and white meat pattern.

To our knowledge, there is only one other study that examined the extent to which socio-economic factors are related to ethnic differences in dietary patterns. According to Sommer et al. (4), SES explained a large proportion of ethnic differences in dietary habits among pregnant women, although the fully adjusted model also pointed to significant cultural differences in dietary preferences. However, no test for effect modification by SES was performed. This insight is important as this could help us understand which subgroups would benefit most from targeted (cultural sensitive) interventions for improving dietary habits.

Within this population of mainly first-generation (85.4 and 89.0% in the South Asian and African Surinamese groups, respectively) Surinamese migrants, education has been primarily completed in Suriname and might, therefore, not be a good proxy of current SES. Educational status has different meanings over the life course and is likely to hold different significance in different countries and cultures (18). Therefore, the level of education might not be an optimal indicator of current SES in the context of dietary behaviour. Occupational status might also act differently among ethnic minority groups (18), their rates of unemployment are higher than in the ethnic Dutch population and, adjusted for characteristics such as educational status or work experience, ethnic minorities do not have comparable chances on the labour market (32). Additionally, income may vary by ethnic group within the same occupational class, so that occupation may not have equivalent meanings across groups (33). Unfortunately, income was not measured in the HELIUS study so we could not explore its influence. More research is needed on useful SES indicators in ethnic minority groups (18, 34). It has been recommended that researchers systematically explore the effect of different SES indicators to demonstrate their cross-ethnic group validity as potential confounding variables for specific groups and outcomes of interest (18).

Some methodological considerations should be addressed. The use of PCA requires several arbitrary decisions about the selection of included variables (FFQ item definition/collapsing foods into food groups), the number of retained factors, and the method of rotation. We conducted sensitivity analyses to investigate whether the higher proportion of ethnic Dutch participants influenced the extracted patterns and found this not to be the case. The labelling of the identified patterns was subjective; this can be judged by the reader from the presented factor loadings (Table 2). Our food groupings were based on our aim of exploring ethnic differences in dietary patterns and followed general-based main analytic decisions on previous scientific knowledge. In addition, there are inherent problems in dietary assessment, such as self-report bias. We used two different FFQs to measure the dietary intake within the ethnic Dutch and Surinamese populations. Differences in pattern scores might be due to differences in the FFQs. However, the FFQs in this study were based on a validated Dutch FFQ (22) and were developed with the aim of conducting combined analyses; therefore, they have the same lay-out, consist of similar, comparable food items, and were developed with the same standardized, rigorously validated methodology (24). The extensive food list of the FFQs used included group-specific marker foods that may be key to elucidating differences between the dietary patterns of the ethnic groups included in this study. Because the response rate of both the Surinamese groups was lower than that of the ethnic Dutch population within the HELIUS-dietary Patterns study (54% versus 79%, respectively), we compared responders with those who did not complete an FFQ. No major differences were observed, except that those who participated were slightly older and higher educated in all three ethnic groups.

The robust ethnic differences in dietary patterns indicate that besides shared characteristics in food group intake, it is also important to account for ethnic differences in the details of dietary behaviour when developing new strategies to promote healthy diets. The absence of a clear SES gradient in the noodle/rice dishes and white meat pattern among both Surinamese groups suggest the importance that is given to the foods characterizing this pattern and underscore the finding of a selective change in dietary behaviour among ethnic minority groups. Thus, the promotion of healthy diets should be based on existing (ethnic-specific) dietary patterns, respecting the value assigned to these patterns. However, as in the host population, promotion of fruit and vegetable intake is particularly relevant for low SES groups.

## Supplementary Material

Socio-economic status and ethnicity are independently associated with dietary patterns: the HELIUS-Dietary Patterns study
