# Supplementary material for: Socio-economic status and ethnicity are independently associated with dietary patterns: the HELIUS-Dietary Patterns study
Source: Food Nutr Res. 2015 Jun 2;59:10.3402/fnr.v59.26317. doi: 10.3402/fnr.v59.26317 (PMC4454783; doi:10.3402/fnr.v59.26317)
Supplement: Socio-economic status and ethnicity are independently associated with dietary patterns: the HELIUS-Dietary Patterns study [file FNR-59-26317-s001.pdf]

Online Supporting Material

Supplemental table 1. Dutch and Surinamese food groups used in the principal component analysis in the HELIUS-Dietary Patterns study

**Alcoholic drinks**	Liquor, Gin, Distilled drinks, mixes (including Breezer)
**Beer**	Beer
**Brassica vegetables**	cabbage raw, cauliflower, broccoli, Brussels sproutsŦ white cabbage, green* cabbage
**Breakfast drink**	Breakfast drink
**Butter**	Butter, herb or garlic butter, low fat butter, butter in cooking
**Cakes and cookies**	Gingerbread, nutritional cookies, muesli/granola bars, cakes and cookies, South Asian sweets* such as barfi, gullab jamun etc.
**Cheese**	All types of cheese, including cheese added to hot meals and as a snack.
**Chicken**	Chicken
**Coffee**	Coffee
**Eggs**	Eggs
**Fat, oil (not olive oil) and full-fat margarine**	Margarine, frying fats/oils, vegetable oils, butter.
**French fries and other fried potato dishes**	Fried potatoes or French fries
**Fruit juices**	Orange juice, other fruit juice without added sugar
**Fruits**	Apples, bananas, oranges, mandarins, grapefruits Ŧ, kiwi, strawberries, grapes, mango*, pineapple*, avocado*, other fruits, applesauceŦ
**High fat fish**	Herring, mackerel, salmon, eel, sardines etc.
**High fibre bread and bread products**	High fibre crackers, bread, whole grain breads, dark bread etc.
**High fibre cereals**	High fibre cereal and cereal products (muesli, oats)
**High fat dairy products**	Whole milk and milk products, full-fat yogurt and yoghurt products (including bioactive yoghurts), full-fat pudding, mousse, custard, quark, ice cream, whipped cream, cream for warm dishes, coffee creamer
**Legumes**	Legumes
**Low fibre bread and bread products**	Low fibre crackers, croissants, white bread and bread rolls, raisin bread
**Low fibre cereals**	Low-fibre breakfast cereals and cereal products
**Low-fat fish**	Tuna, cod, pangasius, trout, tilapia, fish fingers, shellfish etc.
**Low-fat margarine**	Low-fat margarine
**Low fat dairy products**	Low fat milk and milk products (buttermilk, yoghurt drinks, chocolate milk) low-fat yoghurt and yogurt products, low fat quark.

**Meat substitutes and other soy products**	Tofu, tempeh, soya and other meat substitutes
**Nuts and seeds**	Nuts and seeds in warm dishes or as a snack
**Olive oil**	Olive oil
**Organ meat**	Liver, kidney etc. chicken heart*
**Other vegetables**	Raw salad, carrots, cucumber, onions, other raw vegetables, cooked eggplant, courgette, pepper, carrots, green beans, spinach, etc.
**PancakesŦ**	Pancakes
**Pasta**	Whole grain pasta, white pasta
**Pom***	Pom (Surinamese festive dish)
**Potatoes and root vegetables**	Potatoes (boiled, baked, puree), root vegetables *, other than potatoes
**Processed meat**	Salami, ham, bacon, liver sausage, other cold cuts, sausages, etc.
**Roti***	Roti (indian flat bread)
**Red meat**	All types of beef, pork, lamb or other meat as smoked sausage, hot dog sausage
**Rice and noodle dishes**	White/wholegrain rice, fried rice*, noodle (dishes)* etc.
**Savoury bread fillings**	Peanutbutter, sandwich spread Ŧ, fish salad, other salads
**Savoury sauces**	Oils for salads, ketc.hup or other red sauces used by snacks and warm dishes, all types of mayonnaise, sate sauce, other sauces, coconut milk*, soy sauce*
**Snacks**	Fried snacks, croquette, spring rolls, sausage rolls, cheese pastries, shoarma, hamburgers, sate, other warm snacks, chips, other salty snacks as cheese biscuits, crackers with fishŦ, fish saladŦ, pateŦ, cheeseŦ, other toppingsŦ, Bara*, deep fried plantain*, pizza
**Soups**	Soups with legumes, soups with root vegetables*, peanut soup*, and other soups
**Soya-based milk substitutes**	Soya milk, soya yoghurt products, soya ice-cream, soya coffee milk
**Sugar and sweets**	Added sugar or calorie containing sweeteners in cereals, desserts, tea or coffee, ice-lollies, candies, chocolate, sweet bread fillings
**Sugar free/diet drinks**	All types of light/diet soft drinks
**Sugar sweetened beverages**	Fruit drinks with added sugar, cordials, soft drinks, energy and sport drinks
**Tea**	Tea
**Tomato and tomato products (sauces)**	Raw and cooked tomato and tomato saucesŦ
**Water**	Water
**Wine, sherry, port**	Wine, sherry, port vermouth

**vemout**	

Footnote supplemental table 1: *= food item only assessed in the Surinamese FFQ | Ŧ= food item only assessed in the Dutch FFQ. 35 food groups are assessed in a similar way, 11 food groups are assessed in such a way that besides similar food items, also ethnic specific food items were taken into account, 1 food groups was only assessed in the ethnic Dutch FFQ (pancakes), and 2 food groups were only assessed in the Surinamese FFQ (roti and pom).
